# Journalists, district attorneys and researchers: why IRBs should get in the middle

**DOI:** 10.1186/s12910-015-0015-y

**Published:** 2015-03-29

**Authors:** Anna H Chodos, Sei J Lee

**Affiliations:** Division of General Internal Medicine, San Francisco General Hospital, Department of Medicine, University of California, San Francisco 1001 Potrero Avenue, San Francisco, CA USA; Division of Geriatrics, University of California and San Francisco VA Medical Center, 4150 Clement Street, San Francisco, CA USA

## Abstract

**Background:**

Federal regulations in the United States have shaped Institutional Review Boards (IRBs) to focus on protecting individual human subjects. Health services research studies focusing on healthcare institutions such as hospitals or clinics do not have individual human subjects. Since U.S. federal regulations are silent on what type of review, if any, these studies require, different IRBs may approach similar studies differently, resulting in undesirable variation in the review of studies focusing on healthcare institutions. Further, although these studies do not focus on individual human subjects, they may pose risks to participating institutions, as well as individuals who work at those institutions, if identifying information becomes public.

**Discussion:**

Using two recent health services research studies conducted in the U.S. as examples, we discuss variations in the level of IRB oversight for studies focusing on institutions rather than individual human subjects. We highlight how lack of IRB guidance poses challenges for researchers who wish to both protect their subjects and work appropriately with the public, journalists or the legal system in the U.S. Competing interests include the public’s interest in transparency, the researcher’s interest in their science, and the research participants’ interests in confidentiality. Potential solutions that may help guide health services researchers to balance these competing interests include: 1) creating consensus guidelines and standard practices that address confidentiality risk to healthcare institutions and their employees; and 2) expanding the IRB role to conduct a streamlined review of health services research studies focusing on healthcare institutions to balance the competing interest of stakeholders on a case-by-case basis.

**Summary:**

For health services research studies focusing on healthcare institutions, we outline the competing interests of researchers, healthcare institutions and the public. We propose solutions to decrease undesirable variations in the review of these studies.

## Background

Due to several high-profile abuses of human research subjects in the 20th century, the United States Office of Human Research Protections (OHRP) was established to oversee regulations that prevent future abuses. These regulations, widely called the “Common Rule”, guide Institutional Review Boards (IRBs) to review research that involves “human subjects”, which are defined as individuals about whom data is being collected through intervention or interaction with the study or about whom identifiable, private information is obtained [1].

Since OHRP regulations and IRBs focus on human subjects research, health services research focusing on healthcare institutions such as hospitals or health systems have experienced highly variable IRB review of their research. Unlike human subjects research, there are currently no regulations and few guidelines for the review of research focusing on institutions. This may become increasingly problematic in the current era of health care reform within the U.S. as more studies are conducted that focus on health systems. Below, we recount two recent examples of health services research studies which experienced widely divergent levels of IRB review in order to highlight potential competing interests of various stakeholders. Finally, we will conclude with solutions that may help balance these valid competing interests.

## Discussion

### Examples of variation in review of non-human subjects research by IRBs

Study 1: Lagu and colleagues contacted subspecialist practices to request an appointment for a fictional obese and hemiparetic patient, for whom “full and equal access to healthcare services and facilities” is required under the Americans with Disabilities Act (ADA) [2]. Twenty two percent said they were unable to accommodate or see this patient. The IRB chose to review this study. They mandated that the authors contact the practices after the completion of data collection to debrief them and let them know they had been included in a research study. When advised of this research, several practices expressed anger that they had been included in research without their knowledge. *(from personal conversation with Dr. Lagu, permission granted to use)* Furthermore, the IRB instructed the authors to destroy identifying information prior to publication so that individual clinics in violation of the ADA could not be identified [3-5]. After publication, the state Attorney General’s office called Dr. Lagu, asking for identifying information. She informed them that her staff had destroyed the identifying information under the direction of the IRB. *(from personal conversation with Dr. Lagu, permission granted to use).*

Study 2: Rosenthal and colleagues used a “secret shopper” approach to determine the cost of a total hip arthroplasty at twenty top-ranked U.S. orthopedic hospitals (according to the US News and World Report) as well as two randomly selected hospitals from each state. They found that prices were difficult to obtain and when obtained, showed a nearly ten-fold variation between the most-and least-expensive hospitals [6]. Rosenthal and colleagues submitted this study for IRB review, but their IRB declined to review, stating that this was not human subjects research and thus beyond their purview. After publication, the authors were contacted by journalists asking them to identify which hospitals were included from each state and what the costs were with each hospital. They were also contacted by hospital administrators of the twenty top-ranked hospitals asking about the cost quoted by their own hospital as well as the name of the person who provided this information. Without IRB oversight, the researchers released some information on a case-by-case basis. Specifically, they did not identify any individuals who provided cost information, but did provide the cost quoted by a hospital to its own hospital administrators. (*from personal conversation with Dr. Cram, senior author on study, permission granted to use*).

Why were these two studies handled so differently by different IRBs? Should both or neither have been reviewed? Did the two IRBs get it right and only the Lagu study needed review?

### Minimizing risks to non-human subjects

Although neither the Lagu nor Rosenthal study had human subjects, both studies had the potential to expose research participants to risk. If the subspecialty practices included in the Lagu study were identified and fined, the front office staff could have been reprimanded or even fired. Similarly, the hospital staff that provided cost data in the Rosenthal study could also have been disciplined by hospital leadership. Thus, these institutions employed people who could have been harmed via involuntary research participation though they were not the direct subjects of study.

In market research in the United Kingdom, guidelines for “secret shopper” studies recommend that the identity of participants (who are not always consented) should be protected and “the competitor staff or organisation must not suffer any detrimental effect as a result of a mystery shopping exercise” [7]. However, these guidelines are not widely recognized in health services research and without consistent IRB involvement health services researchers must often navigate these complex issues without external oversight.

### Need for guidance in researchers’ interactions with journalists and the legal system

In the Lagu study, if the IRB had not mandated that identifying information be destroyed, what would have been Dr. Lagu’s options when the Attorney General’s office called? Most health services researchers would feel uneasy divulging research data that may put research participants at risk. However, most health services researchers would be uncomfortable declining a request from an Attorney General. The IRB’s foresight in requiring that identifying information be destroyed prior to publication allowed Dr. Lagu to avoid this dilemma. Further, the IRB provided her with support from a system of institutional oversight that insulated her from practices as well as the Attorney General.

Because the Rosenthal study was not reviewed, when they were contacted by journalists asking for the identity of hospitals, they had to decide what to divulge on a case-by-case, ad hoc basis. They did not benefit from an IRB review that could have guided them prior to requests for information from journalists. Their decision to provide limited information in specific cases was very reasonable and their decision to minimize identifying hospitals was probably appropriate. However, additional institutional guidance could have prepared Rosenthal and colleagues and led them to consider the potential risks to their research subjects if the subjects were publicly identified. This in turn would have clarified before publication what information should and should not be divulged to the media. Although the researchers’ approach toward confidentiality was reasonable, they did not explicitly consider the risks and benefits of divulging identifying information prior to being faced with media attention.

### Competing interests of the public, research participants and health services researchers

The U.S. public funds and participates in a substantial portion of research and they have a reasonable expectation to access specific research results. Research subjects often have an interest in maintaining confidentiality of specific information. For example, in the Lagu study, mandating the destruction of information that identifies practices may have been ethical to protect them and the staff that work there. However, by hindering the identification of practices that illegally discriminated against Americans with disabilities, the IRB’s actions left some with the perception that they were more interested in protecting IRB protocols and physician practices than disabled Americans. Paula Span, a New York Times journalist, noted that “shredding the results creates the impression that the IRB values its protocols over the public interest” [8]. Along with the public and research subjects, U.S. researchers have an interest in conducting high-impact research.

The interests from the three stakeholders will often be at odds. Since no clear guidelines for review of non-human subjects research exists, it is often left to the individual researcher to balance these competing interests on an ad hoc basis, as occurred with the Rosenthal study. The history of research abuse suggests that although the vast majority researchers will act appropriately and ethically, few will not. External review is one way to ensure that abuses are minimized.

### Recommendations

To appropriately balance the valid competing interests of the public, non-human research subjects and researchers, we propose the following two recommendations. First, consensus guidelines, for U.S. researchers or more broadly, should be developed that address when external review is necessary and detail how competing interests should be balanced.

Guideline development should engage health services researchers, ethicists, representatives from healthcare institutions, research funders and the public to get a broad-based understanding of the competing interests involved. Ideally, guidelines should start to describe how these competing interests should be balanced. Specific circumstances where the risk of harms are greater (e.g. legal action) should be considered.

Second, IRBs in the U.S. should expand their role to review non-human subjects research. Expanding IRB oversight could be costly and labor intensive, and contribute to increased review burden that already lengthens time from research proposal to active study. However, one middle ground would be to have specialized review sections staffed with health services researchers with experience in non-human subjects research. In most institutions, IRBs are best positioned to provide external research oversight to researchers, given their diverse membership and the similarities of health services research to some types of clinical and medical research.

## Summary

The public supports or participates in most health services research. The public, journalists and the legal sector consume health services research. Health services research is growing as the focus on quality of care and transparency of cost becomes more central to the U.S. health care system. Researchers therefore stand at the interface of health services research, the public and the law. For health services research to continue to grow and thrive, clearer principles should be developed to guide individual researchers and support IRBs in balancing these valid competing interests.
